# Successful Vaginal Delivery Enabled by Continuous Epidural Analgesia for Cancer-Related Pain in Pregnancy: A Case Report

**DOI:** 10.7759/cureus.86283

**Published:** 2025-06-18

**Authors:** Risa Miyazaki, Megumi Kanao-Kanda, Toshiyuki Kuriyama, Sarah K Luthe, Tomoyuki Kawamata

**Affiliations:** 1 Anesthesiology, Japanese Red Cross Society Wakayama Medical Center, Wakayama, JPN; 2 Anesthesiology, Wakayama Medical University School of Medicine, Wakayama, JPN

**Keywords:** cancer-related pain, epidural analgesia, labor positioning, pregnancy, vaginal delivery

## Abstract

Managing cancer-related pain during pregnancy presents a significant clinical challenge due to the limited use of systemic analgesics, such as opioids and nonsteroidal anti-inflammatory drugs (NSAIDs), owing to the potential risks to the fetus. Severe cancer-related pain may impair a parturient’s ability to assume or maintain an optimal labor positioning during labor and delivery, potentially precluding vaginal delivery. In June 2019, a 32-year-old pregnant woman with synovial sarcoma developed intractable gluteal pain that impaired her ability to assume an appropriate delivery position. Given the concerns regarding the use of systemic opioids and NSAIDs, continuous lumbar epidural analgesia was initiated at 34 weeks' gestation to manage cancer-related pain. This approach enabled the patient to tolerate labor positioning, leading to successful vaginal delivery with minimal discomfort. Postpartum pain was managed with oral opioids, and definitive oncologic treatment was initiated one month after delivery. This case demonstrates that continuous epidural analgesia can serve as an effective and safe strategy to manage cancer-related pain during pregnancy, facilitating vaginal delivery when systemic analgesic use is limited.

## Introduction

Severe cancer-related pain during pregnancy can interfere with labor positioning, posing a challenge to achieving vaginal delivery. Systemic analgesics such as opioids and nonsteroidal anti-inflammatory drugs (NSAIDs) are often avoided due to potential adverse fetal effects [1]. Opioids have been associated with neonatal oversedation, respiratory depression, and withdrawal syndrome [2,3], while NSAIDs may cause premature closure of the ductus arteriosus, fetal renal impairment, and delayed labor [4]. Consequently, alternative strategies for pain management are warranted. Here, we present a case in which epidural analgesia provided effective pain relief in a patient with synovial sarcoma during pregnancy, allowing for vaginal delivery while avoiding systemic analgesics.

## Case presentation

**Figure 1:**
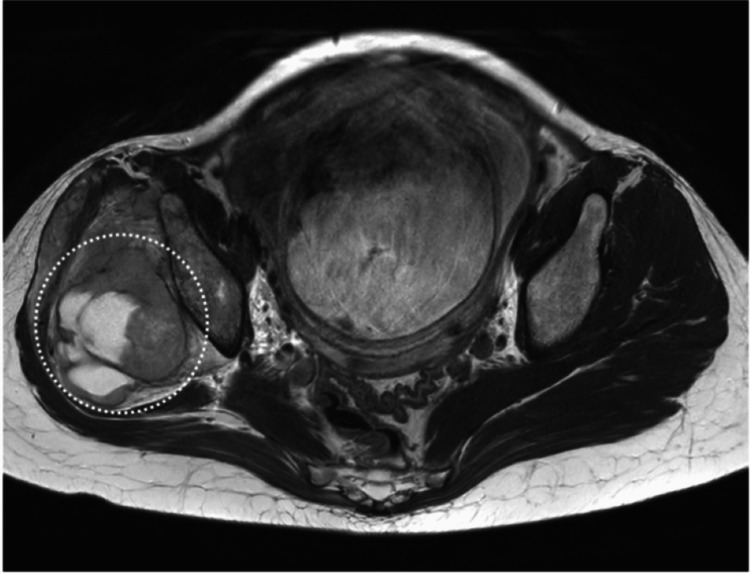
T2-weighted MRI obtained at admission MRI scan showing a large synovial sarcoma (white dotted circle) in the right gluteal region with involvement of adjacent soft tissues.

A 32-year-old pregnant woman with a history of Kawasaki disease presented with right gluteal pain radiating to the lateral thigh, which had been present for two years. The patient had experienced two pregnancies, including one prior successful vaginal delivery, classifying her as a multipara. As the pain was mild and did not interfere with daily activities, the cause of the pain was not investigated until 31 weeks' gestation, when the pain significantly worsened. The pain was initially managed with acetaminophen 2400 mg/day. MRI revealed a tumor involving her right femoral head, and biopsy confirmed the diagnosis of synovial sarcoma (Figure 1).

Due to the urgency of initiating oncologic treatment, labor induction was scheduled at 35 weeks’ gestation.

On June 17, 2019 (at 34 weeks and six days’ gestation), the patient presented with severe, electric shock-like pain in the right L1-4 region, rated 8/10 on the Numerical Rating Scale (NRS), which interfered with sleep, ambulation, and the ability to assume the lithotomy position, the optimal delivery position in her case. An epidural catheter was placed for pain management at the L3/4 interspace. A test dose of 3 mL of 2% lidocaine with epinephrine was administered to confirm catheter placement, resulting in sensory blockade of the L2-4 dermatomes and reducing pain to an NRS of 4/10. Continuous epidural analgesia was initiated using a mixture of 0.8 mg fentanyl, 142 mL of 0.25% levobupivacaine, and 142 mL of normal saline, infused at 5 mL/h.

On June 18, 2019, the patient developed numbness in the left lower extremity, prompting a reduction of the infusion rate to 3 mL/h. Spontaneous labor commenced and was augmented with oxytocin. The patient subsequently delivered vaginally with minimal pain (NRS 1-2/10). The neonate weighed 2072 g with an Apgar score of 10 at 1 and 5 minutes. Postpartum pain was managed with acetaminophen (2000 mg/day) and etodolac (40 mg/day).

On June 19, 2019, the patient reported recurrent pain in the right gluteal region (NRS 6-7/10). Following colostrum expression, the epidural catheter was removed, and oral oxycodone (20 mg/day) was initiated. Continuous epidural analgesia was administered for three consecutive days. During this period, no signs of infection, catheter displacement, or dysfunction were observed, and catheter replacement was not deemed necessary. By June 22, 2019, with the addition of 5 mg rescue doses of oxycodone (2-3 times daily), resting pain decreased to NRS 4-5/10, and the patient was discharged.

Subsequently, neoadjuvant chemotherapy was initiated in July, followed by surgical tumor resection in September, and postoperative chemotherapy in October, which remains ongoing. Written informed consent was obtained from the patient for the publication of this case.

## Discussion

Managing cancer-related pain during pregnancy is particularly challenging, due to fetal safety concerns with systemic analgesics such as opioids and NSAIDs [1-4]. In this case, continuous lumbar epidural analgesia effectively controlled severe synovial sarcoma-associated pain, enabling the patient to assume an appropriate labor position and facilitate vaginal delivery without the use of systemic analgesics.

Synovial sarcoma is a rare soft tissue malignancy that accounts for 5-10% of all soft tissue sarcomas [5,6], with a peak incidence in individuals in their 20s and 30s [7]. Synovial sarcoma frequently arises in the lower extremities and is often associated with significant pain [7]. In our patient, worsening cancer-related pain during late pregnancy led to impaired ambulation and difficulty assuming a delivery position, potentially necessitating cesarean section.

Epidural analgesia provided targeted pain relief while minimizing fetal exposure to systemic drugs, avoiding potential cesarean delivery and possible need for general anesthesia. These interventions are associated with increased maternal morbidity and mortality, with cesarean section carrying a tenfold increase in maternal mortality compared to vaginal delivery [8], and general anesthesia carrying a 16.7-fold higher risk compared to regional anesthesia [9]. Although lidocaine with epinephrine is commonly used as a test dose, clinicians should be aware of the concerns regarding its reliability and safety in obstetric patients, as physiological changes during pregnancy may blunt the expected cardiovascular response, potentially masking intravascular injection [10-12]. Fentanyl-based test dosing has been proposed as a safer alternative in this population [13].

Previous reports have also demonstrated the utility of regional analgesia in similar clinical contexts. For instance, thoracic epidural analgesia has been used to manage thoracoabdominal sarcoma pain in pregnancy while avoiding systemic opioids and achieving favorable maternal and fetal outcomes [14]. Similarly, prolonged epidural catheterization provided effective pain control in a pregnant patient with cervical cancer [15].

## Conclusions

This case demonstrates that epidural analgesia is a safe and effective strategy in selected patients for managing cancer-related pain in pregnancy when systemic analgesic use is limited. By providing adequate pain relief and facilitating appropriate labor positioning, epidural analgesia may help preserve the option of vaginal delivery in this patient population.
